# Computational modelling of aggressive B-cell lymphoma

**DOI:** 10.1042/BST20253039

**Published:** 2025-07-04

**Authors:** Eleanor S. Jayawant, Aimilia Vareli, Andrea Pepper, Chris Pepper, Fabio Simoes, Simon Mitchell

**Affiliations:** Department of Clinical and Experimental Medicine, Brighton & Sussex Medical School, University of Brighton and University of Sussex, Brighton BN1 9PX, U.K.

**Keywords:** cancer, computational biology, computational models, lymphoma, signalling, systems biology

## Abstract

Decades of research into the molecular signalling determinants of B cell fates, and recent progress in characterising the genetic drivers of lymphoma, has led to a detailed understanding of B cell malignancies but also revealed daunting heterogeneity. While current therapies for diffuse large B-cell lymphoma are effective for some patients, they are largely agnostic to the biology of each individual’s disease, and approximately one third of patients experience relapsed/refractory disease. Consequently, the challenge is to understand how each patient’s mutational burden and tumour microenvironment combine to determine their response to treatment; overcoming this challenge will improve outcomes in lymphoma. This mini review highlights how data-driven modelling, statistical approaches and machine learning are being used to unravel the heterogeneity of lymphoma. We review how mechanistic computational models provide a framework to embed patient data within knowledge of signalling. Focusing on recurrently dysregulated signalling networks in lymphoma (including NF-κB, apoptosis and the cell cycle), we discuss the application of state-of-the-art mechanistic models to lymphoma. We review recent advances in which computational models have demonstrated the power to predict prognosis, identify promising combination therapies and develop digital twins that can recapitulate clinical trial results. With the future of treatment for lymphoma poised to transition from one-size-fits-all towards personalised therapies, computational models are well-placed to identify the right treatments to the right patients, improving outcomes for all lymphoma patients.

## Introduction

Lymphoma is a collective term used to describe a heterogeneous range of haematological malignancies, of which diffuse large B-cell lymphoma (DLBCL) is the most common [1]. DLBCL is an aggressive non-Hodgkin lymphoma characterised by vast clinical, molecular and genetic heterogeneity. Despite substantial advances in our understanding of the diverse molecular determinants of DLBCL, the clinical standard of care has remained one-size-fits-all for almost two decades [2]. The first line immunochemotherapy regime R-CHOP (rituximab, cyclophosphamide, doxorubicin, vincristine, prednisolone) effectively cures around 60% of DLBCL patients, while the remaining 40% exhibit either refractory or relapsed disease (R/R-DLBCL) and have very poor prognosis [3]. The subsequent introduction of polatuzamab- or etoposide-containing regimes has shown some promise, but they have not improved overall survival [4] and remain agnostic to the molecular biology of each patient’s disease.

While detailed characterisation of the genetic [5–7], epigenetic [8], protein abundance [9] and tumour microenvironmental heterogeneity [10,11] in DLBCL is now routine, the ability to exploit these data to predictably target the disease has remained elusive. Computational models provide tools to interrogate and integrate multiple data modalities to develop actionable clinical insight. Data-driven approaches, including logistic regression and machine learning (ML), can enable the identification of critical signals in the face of daunting heterogeneous datasets, while mechanism-driven simulation approaches can enable predictive understanding of complex signalling networks and their response to targeted therapies. Thus, leverage of computational models to inform real-time clinical decision making is key to advancing treatment of DLBCL.

### Data-driven approaches

Machine learning, and the statistical regression methods which preceded them, have the power to identify patterns in heterogeneous and noisy data [12]. Applications of ML in healthcare and medical research are rapidly expanding as artificial intelligence techniques have become increasingly accessible. In haemato-oncology, ML is becoming progressively more valuable, particularly in diagnostics and image analysis [12,13].

Predictive prognostic indexes in DLBCL were first developed using regression [14] to identify multiple clinical metrics such as age, cognitive performance, extranodal involvement and lactate dehydrogenase levels in 2124 patients [15], which could be combined to provide reliable prognostic predictions. With increasing amounts of clinical data available for each DLBCL patient, approaches such as least absolute shrinkage and selection operator (LASSO) are required to identify the best parameters for predicting patient prognosis. Clinical variables predictive of outcome have been identified using LASSO, including age, serum albumin levels and red blood cell count. Furthermore, LASSO-developed prognostic predictions can outperform consortium-agreed metrics such as the International Prognostic Index (IPI) and the National Comprehensive Cancer Network IPI (NCCN-IPI) [16].

Application of regression techniques is not restricted to clinical or routinely collected parameters from patient blood, as they can also be applied to gene expression [17] and genetic data [18]. For example, combining RNA-seq and microarray data from multiple patient cohorts and applying LASSO identified gene expression risk signatures that are predictive of outcome [17]. Importantly, when LASSO was applied to gene expression data from 164 formalin-fixed paraffin-embedded (FFPE) tumour samples, it identified prognostically significant patient subgroups independent of IPI and cell-of-origin (COO) [19].

Despite the many analytical developments in the almost 40 years since the conception of LASSO [20], LASSO continues to show exceptional performance for predicting clinical outcome and recently outperformed a random forest ML algorithm in predicting the overall survival of DLBCL patients (training cohort *n* = 848; validation cohort *n* = 363) [16]. Beyond predicting prognosis, LASSO also has the potential to identify the most promising therapeutic targets from differential gene expression analysis. LASSO was used to select genes identified as potential binding sites for a natural product, which were then verified using molecular docking [21]. Applying LASSO to the output from multiple mechanistic simulations has been shown to correctly predict the best targets to experimentally control B cell proliferation [22].

There have been multiple attempts to further develop such scoring approaches using ML, and variable selection methods such as Akaike’s information criterion, to identify the most prognostically informative clinical metrics in DLBCL [23,24]. Recent comparisons of supervised ML algorithms to develop a model of refractory DLBCL identified Naïve Bayes Categorical classifiers as the best-performing model. Through this approach, significant risk factors for refractory DLBCL were identified, including age, IPI score and EBV infection [25]. Interrogating multi-modalities of DLBCL data with sophisticated statistical approaches has advanced our understanding from the traditional two COO subtypes [26,27], to multiple biologically distinct and prognostically predictive molecular subtypes [5–7]. Naïve Bayes classifiers also form the basis of the LymphGen algorithm, which enables classification of DLBCL biopsies into genetic subtypes with distinct prognosis and signalling pathway signatures [7]. Recently, a neural network-based classification tool, termed DLBclass, improved the accuracy of classification of DLBCL based on whole exome sequencing data [28].

There is substantial agreement between different clustering methods, despite the differences in statistical approaches [29]. For example, the C1 cluster identified by Chapuy *et al*. [5] is enriched with NOTCH2 mutation and BCL6 fusion and significantly overlaps with the BN2 subgroup identified by Staut and colleagues [30]. As such, these clusters align with biological mechanisms (e.g., nuclear factor-κB [NF-κB] signalling activation is prevalent in the MCD-Staut and C5-Chapuy clusters), and therefore, different clusters have the potential to be vulnerable to different targeted therapies. However, clinical trial design has largely ignored DLBCL subtype and not been designed to observe responses to treatment in specific subgroups [29]. Retrospective analysis of the ‘Phoenix’ trial, which treated non-germinal centre (GC)-DLBCL patients with R-CHOP with and without ibrutinib, revealed significantly improved prognoses for patients with the MCD and N1 subtypes following treatment with ibrutinib [31].

### Mechanistic modelling

**Figure 1:**
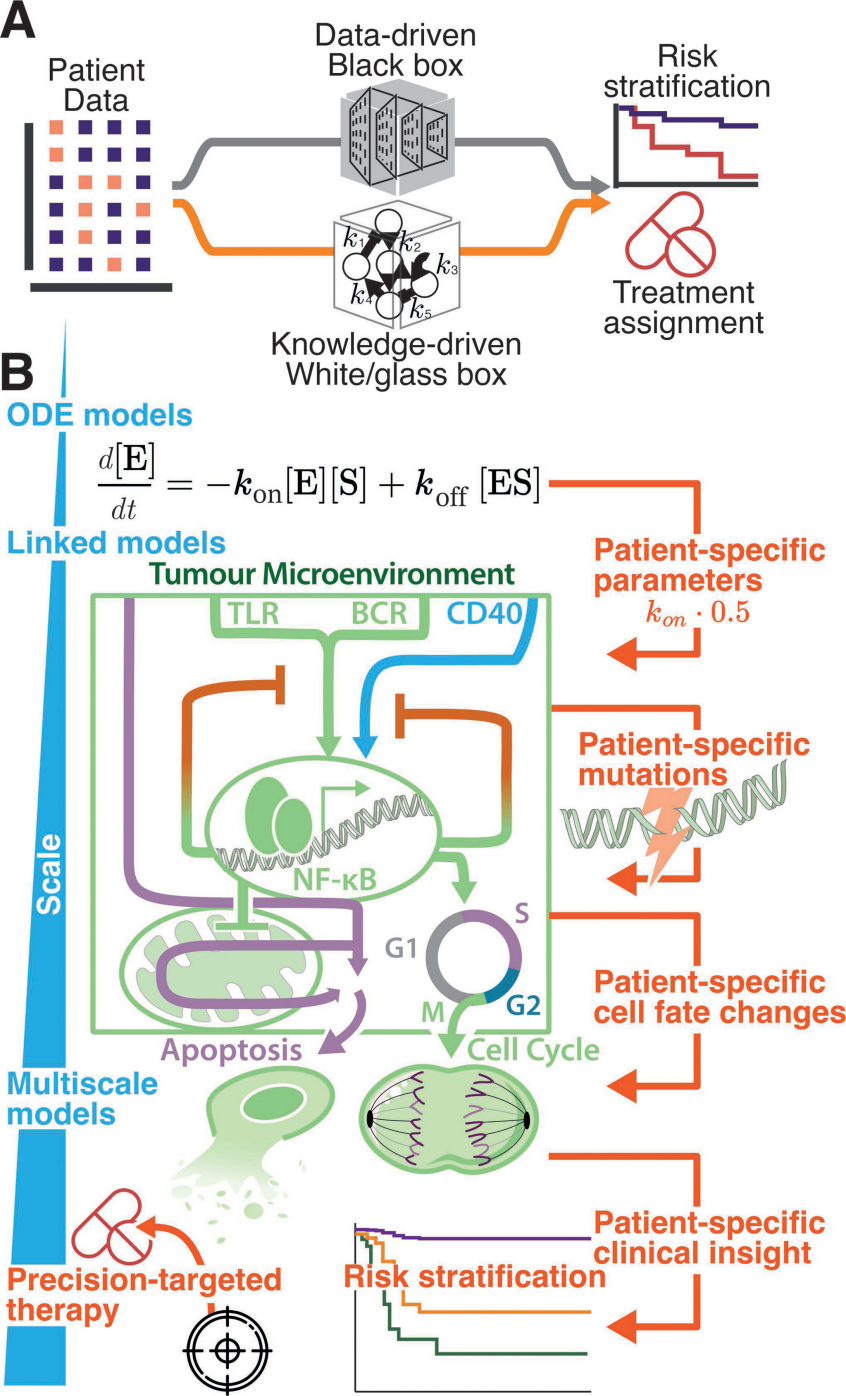
Mechanistic systems biology modelling embeds knowledge to enable predictions across scales (**A**) Data-driven approaches and knowledge-driven approaches both can use patient data to stratify risk and assign appropriate treatments to groups of patients. Both approaches produce valuable outputs, but the ‘black box’ in the data-driven approach may provide limited insight into how predictors and outcomes are related. (**B**) An example of a knowledge-driven approach. Ordinary differential equation (ODE)-based models can be customised with patient-specific parameters, mutations or cell fate changes, or linked with other ODE models to produce multiscale models in which cells can react to microenvironmental stimuli (such as TLR, BCR and CD40L), divide and undergo apoptosis. These approaches can provide patient-specific insight and personalised medicine.

At their core, ML, regression, and LASSO are techniques that are designed to learn how genetic, molecular, and clinical data map to disease characteristics. While they have striking power, frequently the trained models are a ‘black box’, providing limited insight into how predictors and outcomes are related (Figure 1A, top) [12]. Importantly, each new application starts with a clean slate, and without leveraging many decades of detailed characterisation of healthy and malignant B cell signalling and cell fate control.

Mechanistic models provide an alternative approach, grounded in knowledge encoded in their interactions and parameters [32]. In addition, results from mechanistic models can be fully interpretable, mirroring an experiment in which every molecular concentration or clinical parameter can be simultaneously monitored. This ‘white/glass box’ property can enable computational predictions to be more easily validated in the lab or in a clinical trial (Figure 1A, bottom; Figure 1B). Progress in mechanistic modelling of aggressive lymphoma has mirrored biological and clinical progress. Just as the COO lymphoma subtypes were defined through alignment with healthy B cell types [33], mechanistic models of B cell lymphoma are based on models of healthy B cells [34–37]. Similarly, as aberrant NF-κB activation and up-regulation of anti-apoptotic BCL2-family proteins were being clinically and experimentally implicated in poor prognosis DLBCL [5], mechanistic models of NF-κB and apoptosis were reaching maturity [34,38].

### Modelling NF-κB in lymphoma

The NF-κB signalling pathway plays a crucial role in many biological processes including inflammation, cell proliferation and survival, and apoptosis [39]. Dysregulation of NF-κB is a major driver in the pathogenesis of many haematological malignancies, including lymphoma, where it initiates and maintains an anti-apoptotic and pro-proliferative gene expression programme [40]. NF-κB signalling spans two pathways, the canonical and non-canonical signalling pathways, utilising five different subunits, which can form 15 potential homo- or heterodimers [41]. Among the numerous NF-κB target genes are multiple inhibitors of NF-κB (IκBs) creating feedback loops that dynamically regulate NF-κB. This complexity and heterogeneity makes computational models of NF-κB particularly valuable for understanding the regulation of NF-κB in healthy B cells and its dysregulation in disease.

The complexity of NF-κB signalling is further compounded in lymphoma by a combination of recurrently occurring activating mutations and interactions within the tumour microenvironment (TME). There is an emerging picture of distinct roles for canonical and non-canonical NF-κB [42], and even individual NF-κB subunits [32,42,43]. Although the roles of the TME remain poorly understood, it clearly contributes to tumour development and survival and is therefore an important therapeutic target [44,45]. Non-malignant cells in the TME are known to promote tumourigenesis and metastasis, often through activating NF-κB signalling [46]. CD40 in the TME of DLBCL is prognostically predictive and known to activate the non-canonical pathway [47]. Recent single-cell RNA-seq has identified the main signalling from the TME to DLBCL results from CD40L, expressed by T cells; and BAFF, secreted by myeloid cells and cancer-associated fibroblasts [48]. Neutrophil extracellular traps have been found to activate canonical NF-κB signalling in DLBCL cells through Toll-like receptors (TLR), promoting DLBCL progression [49].

Detailed computational models of NF-κB signalling have been iteratively developed and validated with experimental work over the last two decades, particularly in the context of innate immune signalling [50]. These are signalling network models, in which ordinary differential equations (ODE) represent the negative feedback loop between NF-κB and its inhibitors. Such models have revealed discrete functional roles for the inhibitory proteins in NF-κB regulation, which have been experimentally validated. For example, IκBε and IκBδ have been implicated in cRel-specific signalling attenuation and suppression of sustained NF-κB activation, while IκBα has been identified as sufficient for the first peak of NF-κB activity [51–53]. IκB loss by inactivating mutations can induce constitutive NF-κB activation in lymphoma [54] and is associated with drug resistance [55].

The scope and detail of NF-κB models have been iteratively expanded [50,53,56–58]. To date, the most detailed models of NF-κB signalling have focused on the interplay between IκBs and NF-κB subunits, although other models have accurately captured the molecular detail between cell surface receptors such as TLRs and the B cell receptor (BCR) [32,59]. This work has culminated in computational models of NF-κB signalling that integrate the main components that are commonly dysregulated in poor prognosis DLBCL patients (e.g. CD79B and CARD11 within BCR signalling, MYD88 within TLR signalling, and NFKBIE and TNFAIP3 within core negative regulation of NF-κB) [60,61]. Due to its well-characterised role in driving lymphoma pathology, most mechanistic models of lymphoma encode regulation of a single NF-κB component by cell surface receptors such as CD40 and BCR signalling [37,62]. It is becoming increasingly clear that RelA, cRel and RelB are heterogeneously abundant between patients [34,63–65]. However, this detail is not captured by mechanistic models of lymphoma that represent all NF-κB signalling as a single component of the model [37,62], and coarse-grained models of NF-κB may not sufficiently capture NF-κB heterogeneity in DLBCL [32].

Recently, we investigated the heterogeneous composition of NF-κB in DLBCL using a combination of experimental methods and linking of previously described computational models [59,66,67]. This revealed heterogeneity in NF-κB signalling states beyond COO subtypes. We assessed the effect of TLR signalling in the DLBCL TME [68] using flow cytometric data collected for healthy B cells and DLBCL cell lines and primary patient cells. Experimentally derived abundances of RelA, cRel and RelB were used to create computational models of virtual DLBCL cells by manually adjusting the NF-κB subunit synthesis rates to recapitulate experimentally observed NF-κB subunit abundance. These virtual cell models predicted that while high RelA expression was predicted to confer increased RelA:p50 response to the TME, NF-κB-activating mutations can render RelA:p50 insensitive to TME-mediated activation, a prediction that was experimentally validated.

Given its pivotal role in the pathology of lymphomas, NF-κB has long been considered to be a promising therapeutic target. However, the use of broad NF-κB inhibitors in the clinic caused severe on-target toxicities [69,70]. More recently, progress has been made in the development of inhibitors that target specific NF-κB components [71–73]. Understanding the heterogeneity of NF-κB and the response to the TME in DLBCL is vital in order to improve treatment outcomes with these next-generation targeted NF-κB inhibitors. This is likely to require pre-therapy stratification of patients to identify the best NF-κB subunit to target for each individual patient [68].

Targeting the non-canonical NF-κB signalling pathway offers an alternative approach that may overcome the toxicity associated with canonical pathway inhibitors. Computational models have highlighted the important role of the non-canonical NF-κB protein RelB in B cell maturation [52], and a subset of DLBCL patients show elevated RelB signalling [42]. Models have also been central in identifying crosstalk between canonical and non-canonical NF-κB pathways [52,74,75]. cRel was historically associated with canonical NF-κB signalling, but it is now recognised that it is involved in cross-talk between the two pathways. Indeed, computational models predict that chronic activation of NF-κB, as seen in many lymphomas, may prime cRel to respond to non-canonical stimuli. This is significant, as aberrant stimulation of cRel is oncogenic and implicated in a subset of lymphoma [32,40]. As detailed NF-κB models, designed to understand immune signalling, are applied to lymphoma, they are enabling a new level of understanding of the complex signalling dysregulation that leads to cancer.

While chemoimmunotherapy treatments such as R-CHOP form the basis of treatment for DLBCL, radiotherapy is used as a salvage or bridging treatment before stem cell transplantation or chimeric antigen receptor (CAR) T-cell therapy [76]. The DNA damage induced by both treatment options activates NF-κB, providing a mechanism by which lymphoma cells may resist cell death [77]. A computational model of DNA-damage induced NF-κB activation reveals poly ADP-ribose polymerase (PARP) inhibition as a promising strategy to overcome this effect [78]. Targeting specific activated NF-κB components with inhibitors is another potential option.

### Modelling B cell fate decisions

NF-κB activation contributes to lymphoma through its induction of pro-proliferative and anti-apoptotic target genes leading to an imbalance between cell division and death. Computational models of apoptosis have evolved since the early 2000s with increasing detail and validation in multiple contexts [79–83]. More recently, models have revealed how the selective interactions of BCL2-family proteins control apoptosis in cancer and response to chemotherapy [84–86]. Models of the cell cycle have progressed similarly, initiating in xenopus oocytes and fission yeast [87,88], before being extended and applied to cancer [89,90]. Multiple NF-κB target genes are present in models of apoptosis and the cell cycle, including MYC, BCL2, BCLXL and CCND1, which enables models of NF-κB signalling [51] to be linked with models of apoptosis [83] and the cell cycle [89] in order to produce an agent-based multiscale model in which B cells can divide and die. This work enables signalling changes to predict cell fates and cell population changes and revealed how cRel protects growing cells from apoptosis [91].

Leveraging modelling to inform our understanding of B cell lymphoma and rational assignment of therapy requires B cell fates to be predictable and not dominated by unpredictable molecular noise. As stochastic models were able to explain cell population changes caused by increases in MYC and loss of BCL2 in transgenic mouse B cells [92,93], the prevailing view for many years was that cellular proliferation in B cells was a stochastic process. However, single cell lineage tracking experiments, combined with mathematical models, revealed that B cell fate decisions are non-stochastic and can be reliably predicted from molecular signalling in the founder cell. The predictability of cell fates using modelling demonstrates the potential to reliably inform the use of targeted inhibitors in lymphoma. As Blimp1 and IRF4 are also NF-κB target genes and play a key role in B cell terminal differentiation, Roy *et al*. [34] explored the role of cRel and RelA in a model of antibody-secreting cell (ASC) differentiation [94]. Although cRel drives B cell proliferation as previously identified, RelA is necessary for ASC differentiation by inducing Blimp1, which in turn represses cRel [34]. Germinal centre B cells depend on elevated cRel for proliferation, while terminally differentiated ASCs (plasma cells and plasmablasts) undergo cRel down-regulation leading to RelA being the predominant NF-κB subunit. As activated B cell (ABC)-DLBCL cells are defined by a transcriptional signature similar to B cells poised for terminal differentiation, the distinct roles for RelA and cRel seen in healthy B cells are consistent with the emerging understanding of subunit-specific roles in lymphoma, with cRel particularly implicated in GC-DLBCL [40].

### Patient-specific models in lymphoma

Due to the vast heterogeneity of lymphoma and the differential responses to treatment, it is important to better understand differences between patients. Patient-specific models provide the opportunity to characterise lymphoma patients and have been previously used with promising results in other cancers [95,96], including acute myeloid leukaemia [97].

DLBCL patients on average harbour 7–17 driver mutations, in up to 150 recurrently mutated genes [5]. Logical modelling, in which each modelled biomolecule can have a number of discrete states (i.e. low, medium and high activity/expression), has been employed to create patient-specific simulations from whole exome sequencing data. Thobe *et al*. [37] analysed patient data for copy number alterations and mutations, which were incorporated into models as either loss-of-function or gain-of-function, with component values set to 0 or 1 respectively. This resulted in a unique model for each patient, with differences in the states of components based on the individual patients’s mutational profile. Logical patient-specific models created in this way revealed a large variety of oncogenic states and enabled patients to be stratified according to predicted dependency on NF-κB signalling. Simulating targeted inhibitors in these patient-specific models predicted that different patients could have highly variable responses to the same inhibitors [37].

While significantly more computationally challenging, continuous models enable each simulated biomolecule to have any value of activity/abundance. These models, such as the linked mechanistic model described above [34], can capture emergent effects resulting from mutations that cannot be captured by logical models. These models contain many of the genes, or signalling molecules affected by recurring mutations in DLBCL. Norris *et al*. [35] used this model [34] to assess the impact of co-occurring mutations on cell fates in DLBCL and multiple myeloma patients. Simulating the individual effect of BCL2 and MYC mutations had a limited impact on apoptosis and cell cycle, respectively. However, when combined in a multiscale model of double hit lymphoma (co-occuring BCL2 and MYC dysregulation), the model predicted a dramatic alteration in B cell fates, effectively recapitulating clinical trial data. Mutation profiles from whole exome sequencing of DLBCL were filtered using OncoKB based on likelihood of oncogenicity and incorporated into simulations by mapping to modelled parameters [98,99]. Parameters affected by gain- or loss-of-function mutations were increased or decreased by 50%. This approach resulted in patient-specific models that differed only by parameters affected by mutations. The personalised simulations identified subgroups of patients with simultaneous anti-apoptotic and/or pro-proliferative signalling signatures that did not align with COO or genetic clustering. Stratifying patients based on these subgroups generated striking prognostic predictions, which were further enhanced when combined with clinical metrics such as IPI, stage or genetic cluster [35]. The findings were validated in two further patient cohorts [6,100]. As such, this study demonstrated how embedding mutational data within extensive quantitative knowledge of molecular signalling can transform the power of this data.

Quantitative systems pharmacology approaches have also been used to create patient-specific models of lymphoma. This modelling approach shifts the focus from molecular signalling to immune cell composition, cytokine expression and interaction with tumour cells [101]. As such, these approaches are valuable tools in predicting response to cell-based therapies. They have been applied to mitigate the risk of cytokine release and identify predictive biomarkers of response in a clinical trial of CD20/CD3 bispecific antibodies [101,102].

Taken together, patient-specific models that include mechanistic detail of molecular signalling have demonstrated potential to improve the prediction of outcomes to immunochemotherapy [35], and pharmacology models have been shown to predict response to cell-based therapies [102]. While this makes both approaches valuable for risk-stratification of future clinical trials, the true power of modelling in DLBCL may be in identifying the right treatment or combination of treatments for each patient.

### Modelling targeted therapies in lymphoma

One-size-fits-all immunochemotherapy leads to R/R-DLBCL in a substantial proportion of patients [103]. Small molecule inhibitors, targeting specific proteins or their interactions, have revolutionised the treatment of related malignancies such as chronic lymphocytic leukaemia (CLL) and multiple myeloma (MM) [104–107]. However, patient-to-patient heterogeneity has precluded the successful deployment of targeted inhibitors in DLBCL, despite multiple large clinical trials [31,108,109]. Repeatedly, trials of targeted therapies have retrospectively found subgroups of patients that responded but attempts to prospectively use tumour biology to assign therapy have failed [110]. Computational modelling has the potential to overcome this longstanding challenge.

BH3-mimetics, a class of drug that function as competitive binders to BCL2-family proteins, are one such drug. BH3-mimetics have been highly effective in CLL but have not been effectively deployed in DLBCL. Using a mechanistic model of BCL2-family protein interactions at the mitochondrial membrane and simulating the effect of BCL2, BCLXL and MCL1 inhibition in a heterogeneous population of virtual cells, Cloete *et al*. accurately predicted differential *in vitro* responses to BH3-mimetics [111]. This work identified the importance of considering genetic mutations in virtual lymphoma; the correlation between computational prediction and experimental data was enhanced when genetics were used to inform the model. This reinforces the previously discussed finding that embedding mutations in models is necessary to accurately predict DLBCL response to the TME [68].

Due to the complex signalling dysregulation in lymphoma, it is unlikely that any single targeted therapy will be highly effective in many patients. Therefore, much computational modelling has focused on identifying synergistic combinations of targeted therapies. Cloete *et al*. simulated combinations of BH3-mimetics and identified a cell line-specific synergistic interaction between BCL2 inhibition and MCL1 inhibition that was computationally predicted and experimentally validated [111]. The effect of BCL2 inhibitors alone and in combination with BTK, PI3K or NF-κB inhibitors has also been modelled in patient-specific simulations, showing heterogeneous responses based on NF-κB signalling states [37]. A mechanistic model, focused on signalling downstream of the BCR (including NF-κB, ERK, and AKT), was used to simulate combination therapies which were then ranked according to predicted synergy. Synergistic and antagonistic pairwise combinations were predicted and validated experimentally [62].

While synergy metrics, such as Bliss independence [112], are an attractive method to identify promising drug combinations, there is evidence that the most important property of combination therapies is non-overlapping resistance mechanisms (i.e. resistance to one drug does not confer resistance to the other) [113]. Indeed, a detailed dissection of the drugs combined in R-CHOP, using combined CRISPR screening and clonal tracking, revealed that the components of R-CHOP do not combine synergistically but in fact are independently effective with non-overlapping resistance mechanisms [113]. Representing individual patients using unique distributions of drug sensitivities and quantifying the effect of non-overlapping resistance mechanisms is also able to explain clinical trial results of targeted inhibitors added to R-CHOP [114].

These studies show that the outcomes of targeted therapies, and therapy combinations, on lymphoma cell lines and patients can effectively be predicted using modelling approaches. Additionally, models of CHOP and R-CHOP have been used to identify the optimal number of treatment rounds in patients [115,116].

### Conclusions and outlook

A predictive understanding of signalling in DLBCL is necessary to advance treatment options, and computational models provide a way to integrate our knowledge of signalling pathways, TME-mediated signalling activation and genetic lesions. Models can integrate this information in a cell line-specific or patient-specific manner. While this review focuses on lymphoma, it is likely that detailed models of signalling, such as NF-κB or apoptosis, will provide insight into other haematological malignancies and cancers more broadly. Computational modelling is becoming increasingly accessible in the digital age, particularly with the rapid increase in the exploitation of artificial intelligence and ML techniques in research. Additionally, the abundance of vast datasets accessible via tools such as cBioPortal and DepMap allows a computational biologist to leverage existing data to rapidly develop and validate models of lymphoma without setting foot in a laboratory.

While data-driven models such as LASSO and ML classifiers have shown impressive predictive power for patient prognosis in lymphoma, such approaches often act as ‘black boxes’, limiting biological interpretability [12]. In contrast, mechanistic models, grounded in biochemical kinetics and systems biology, offer explainable, hypothesis-driven insights into cellular signalling [32]. Data-driven and mechanistic strategies can be complementary, as data-based approaches are highly scalable while knowledge-driven approaches allow insight into molecular mechanisms. LASSO and related ML techniques can be used to inform and constrain mechanistic models; for example, a combined LASSO and ODE modelling approach has recently been used to explore cancer lineages from subclonal populations [117]. One intuitive combination of approaches is to leverage ML to stratify patients based on all available clinical data in order to predict the risk of poor outcome and then leverage mechanistic simulations to identify therapeutic vulnerabilities within at-risk patient groups.

The development of ‘digital twins’, utilising patient-specific models to allow clinicians to stratify patients according to risk, may also predict the most effective treatments for individual patients (Figure 1B). Although the outlook remains promising, as models of patient-specific outcomes and responses to treatment are rapidly advancing, there is still a significant gap between model predictions and the deployment of personalised therapies in the clinic. Clinical trials often involve R-CHOP combined with a targeted inhibitor [118]. Therefore, it is important that models can effectively capture combined responses to both multi-drug immunochemotherapy regimens and targeted therapies in order to effectively predict clinical trial outcomes. As models continue to improve, computational modelling will likely inform clinical trials through risk stratification and treatment assignment.

Reconciling the multiple sources and scales of heterogeneity remains incomplete. As multiscale models span the temporal scale from minutes to years, the relevance of each source of variability changes. While on the timescale of hours to days, cell fates are reliably inherited, slight molecular heterogeneity introduced during cell division can accumulate. Computational modelling indicates this can result in clonally related B cells recreating molecular heterogeneity observed between unrelated cells over approximately 30 cell divisions. Therefore, it is likely that molecular cell-to-cell signalling heterogeneity is not relevant over clinically significant timescales. This is supported by the ability of computational models that omit cell-to-cell heterogeneity to predict patient responses over decade-long timescales using only patient genetics, which are relatively far more stable [35,119]. Studies demonstrating the prognostic power of TME profiling also suggest that patient-to-patient heterogeneity in microenvironmental composition may be incorporated into these modelling frameworks as a deterministic process with predictable influences on lymphoma [120].

The clear challenge for the field is to use computational models to assign effective therapies prospectively in a clinical trial that demonstrates superiority of this approach over a one-size-fits-all approach. Funding for such a trial is frequently challenged by the business model of pharmaceutical companies, which generally prioritises large all-comers design of novel therapies, disincentivises patient stratification into smaller cohorts, and disincentivises drug repurposing of therapies that are not efficacious when given with a one-size-fits-all approach. Affecting such a significant change will be challenging, but repeated failure of biologically-agnostic trial design will increasingly motivate risk-stratified, precision-targeted, and personalised trials. The foundation has been established with the literature described here, and we believe that the development and deployment of computational models are necessary to unlock improved outcomes in lymphoma. It is vital that patients remain at the heart of lymphoma research, both experimental and computational. Treating more patients with targeted therapies, enabled by computational modelling, will also ensure the minimum possible treatment-related adverse events and the highest chance of treatment success.

**Perspectives**

- In recent years, there has been groundbreaking multi-omic data generated, revealing vast patient-to-patient heterogeneity in B lymphoma. New approaches are needed to integrate the emerging data with established knowledge of B cell signalling health and disease.
- Here we review progress in using computational modelling to understand and better treat lymphoma, with a focus on diffuse large B cell lymphoma (DLBCL). We discuss how statistical modelling approaches are identifying patterns in heterogeneous data. We review the development of regression and machine learning approaches applied to multi-modal lymphoma data. We then focus our review on how mechanistic modelling is rapidly revealing patient-specific outcome predictions and, enabled by its encoding of existing knowledge, is uncovering promising tailored therapeutic approaches in DLBCL.
- We argue that the failure of multiple clinical trials to prospectively match each lymphoma patient’s disease biology with effective targeted therapies motivates new approaches to clinical trial design and clinical trial funding. We argue that progress beyond one-size-fits-all treatments in lymphoma requires computational modelling to be embraced. We discuss how using computational modelling approaches to get the right treatments to the right patients will improve patient outcomes.

## Data Availability

No new data were generated for this article.

## Abbreviations

COO: cell-of-origin
DLBCL: diffuse large B-cell lymphoma
IPI: International Prognostic Index
LASSO: least absolute shrinkage and selection operator
ML: machine learning
